# Profiling of Chlorogenic Acids from *Bidens pilosa* and Differentiation of Closely Related Positional Isomers with the Aid of UHPLC-QTOF-MS/MS-Based In-Source Collision-Induced Dissociation

**DOI:** 10.3390/metabo10050178

**Published:** 2020-04-29

**Authors:** Anza-Tshilidzi Ramabulana, Paul Steenkamp, Ntakadzeni Madala, Ian A. Dubery

**Affiliations:** 1 Research Centre for Plant Metabolomics, Department of Biochemistry, University of Johannesburg, Auckland Park 2006, South Africa; 201404841@student.uj.ac.za (A.-T.R.); psteenkamp@uj.ac.za (P.S.); 2 Department of Biochemistry, University of Venda, Thohoyandou 0950, South Africa

**Keywords:** *Bidens pilosa*, cell culture, chlorogenic acids, hydroxycinnamic acids, ISCID, metabolomics, phytochemicals

## Abstract

*Bidens pilosa* is an edible herb from the Asteraceae family which is traditionally consumed as a leafy vegetable. *B. pilosa* has many bioactivities owing to its diverse phytochemicals, which include aliphatics, terpenoids, tannins, alkaloids, hydroxycinnamic acid (HCA) derivatives and other phenylpropanoids. The later include compounds such as chlorogenic acids (CGAs), which are produced as either *regio*- or geometrical isomers. To profile the CGA composition of *B. pilosa*, methanol extracts from tissues, callus and cell suspensions were utilized for liquid chromatography coupled to mass spectrometric detection (UHPLC-QTOF-MS/MS). An optimized in-source collision-induced dissociation (ISCID) method capable of discriminating between closely related HCA derivatives of quinic acids, based on MS-based fragmentation patterns, was applied. Careful control of collision energies resulted in fragment patterns similar to MS^2^ and MS^3^ fragmentation, obtainable by a typical ion trap MS^n^ approach. For the first time, an ISCID approach was shown to efficiently discriminate between positional isomers of chlorogenic acids containing two different cinnamoyl moieties, such as a mixed *di*-ester of feruloyl-caffeoylquinic acid (*m*/*z* 529) and coumaroyl-caffeoylquinic acid (*m*/*z* 499). The results indicate that tissues and cell cultures of *B. pilosa* contained a combined total of 30 *mono*-, *di-,* and *tri*-substituted chlorogenic acids with positional isomers dominating the composition thereof. In addition, the tartaric acid esters, caftaric- and chicoric acids were also identified. Profiling revealed that these HCA derivatives were differentially distributed across tissues types and cell culture lines derived from leaf and stem explants.

## 1. Introduction

*Bidens pilosa* L. (*B. pilosa*) is a flowering edible herb from the family Asteraceae, commonly known as “Blackjack” and as “Beggar’s tick” or “Needle grass” and has been reported to be a nutritious food and medicine source [1,2,3]. This plant is thought to have originated from South America but has spread to most tropical hot areas including African countries [4]. *B. pilosa* has been shown to treat over 41 diseases and conditions [2], such as malaria [5], diabetes [6], hypertension [4], obesity [7] and syphilis [8]. *B. pilosa* has various bioactivities owing to its diverse phytochemical constituents, of which an estimated >300 have been identified [2,3]. These include a variety of aliphatics, terpenoids, phenylpropanoids, tannins, alkaloids, cardiac glycosides, and porphyrins [9,10].

Plant secondary metabolites are important in the production of flavors, fragrances, pharmaceuticals, food additives, and many other applications [11,12]. Therefore, ways to produce important secondary metabolites effectively at a large scale are needed. However, plants as a source of these metabolites may have slow growth or produce metabolites at low concentrations [13,14]. Extracts from whole plants may also pose environmental threats such as reduction of local plant populations and loss of plant genetic diversity [11,12]. A variety of environmental factors (soil fertility, salinity, temperature, light, and soil water) can result in fluctuations in plant secondary metabolites which can lead to significant changes in the plant’s phytochemical composition [15]. Plant cell culture has become an attractive approach as it permits synthesis of secondary metabolites produced in parent plants independent of geographical and seasonal factors [13,16]. Callus culture is initiated on semi-solid media from explant material and this is then used to subsequently initiate cell suspensions with high rates of cell multiplication [17]. Cell suspension cultures permit superior homogeneity of treatments compared to plant tissues and shorter condensed biosynthetic cycles and also permits scaling up of secondary metabolite production in appropriate bioreactors [18,19]. Moreover, a range of chemicals produced by parent plants can also be reproduced in cell cultures as a result of plant cells being totipotent and their retention of the plant’s genetic information [11,17]. 

Chlorogenic acids (CGAs) are a group of esters between hydroxycinnamic acids (HCAs), such as caffeic acid (CFA), ferulic acid (FA), *p*-coumaric acid (*p*-CoA), and sinapic acid (SA) to quinic acid (QA or 1L-1(OH),3,4/5-tetrahydroxycyclohexane carboxylic acid) [20,21,22]. CGAs are complex molecules exhibiting different physiochemical properties due to positional esterification on the quinic acid moiety, leading to *regio*-isomers. Major groups of CGAs are the *mono*- and *di*-caffeoylquinic acids (CQAs) [23]. Furthermore, the basic CGAs can be divided into several groups, based on the number and position of the acyl groups attached to the QA, as *mono*-, *di*-, *tri*-, and *tetra*-esters [20]. The complexity of these acids is vast as mixed *di*-esters such as caffeoylsinapoylquinic acid [22] and caffeoylferuloylquinic acid are found [24]. In addition, a fraction of the naturally occurring *trans*-isomers can occur as *cis*-isomers [25]. Naturally, plants produce these derivatives in response to abiotic stress to aid in the protection of the plant [26,27]. They can also be produced in response to biotic stress, as CGAs have been shown as effective defense phytochemicals against several insect herbivores [28]. 

Identification and quantification of structurally related compounds is challenging and may require the use of reliable advanced analytical techniques. In previous studies, geometrical isomers of CGAs have been differentiated using elution order, but this has been shown to be unreliable [23], mainly due to differences in column chemistries [29]. Discrimination between geometrical isomers has been achieved by liquid chromatography-mass spectrometry (LC-MS) through the use of in-source collision induced dissociation (ISCID), where *cis*-isomers of *di*-CQAs were shown to preferentially bind to alkali metals (sodium, lithium, and potassium adduct-MS), differentiating them from *trans*-isomers [30].

Development of LC-ion trap (IT)-MS-based hierarchical keys for the differentiation of acyl-quinic acids using mild fragmentation conditions has allowed for the confident assaying of most CGA *regio*-isomers using their MS^n^ fragmentation patterns [31,32]. Other advanced technologies such as ion mobility mass spectrometry-MS (IM-MS) allow for the differentiation of positional isomers of CGAs and drift tube ion mobility spectrometry-mass spectrometry (DTIMS-MS) for the elucidation of *cis/trans*-isomers of *di*-CQAs [23]. 

Quadrupole time-of-flight mass spectrometry (QTOF-MS) has also been applied for the differentiation of *regio*-isomers of CGAs [33]. However, more innovations are required for the full characterisation of these metabolites and their isomeric forms. Recently, a QTOF-MS-based ISCID approach has been used successfully to distinguish between CGA *regio*-isomers of both *mono*- and *di*-acylated quinic acids [34,35]. ISCID is proposed as an alternative to multistage MS, whereby precursor ions are initially isolated in the mass analyzer where fragmentation of multiple precursors are acquired simultaneously, similarly to MS^E^ [36,37]. Fragmentation occurs under an intermediate pressure (between high pressure and high vacuum) and applied voltages accelerate ions, promoting collision with the surrounding gas. This permits multiple activation–fragmentation episodes before ions reach the mass analyzer, allowing for efficient structural elucidation [36,37]. ISCID proves to be superior to MS^E^, as in this method in-source dissociation is performed prior to regular MS/MS. This then emulates pseudo-MS^3^, acquiring fragmentation patterns typical for IT mass spectrometers [38].

In the present study, an optimized ISCID method was used, in combination with metabolomic tools and approaches, to differentiate between CGAs *regio*-isomers (including *di*-esters) in tissues, callus and suspension-cultured cells of *B. pilosa*. The results of the current study also contribute to the profiling of CGAs in differentiated tissues vs. undifferentiated cells and contribute to possible identification of any underlying biochemical mechanisms with regard to CGA biosynthesis within tissues and cell cultures of *B. pilosa*.

## 2. Results

### 2.1. Profiling of Chlorogenic Acid Derivatives in Tissues and Cell Cultures of *B. pilosa*

**Figure 1:**
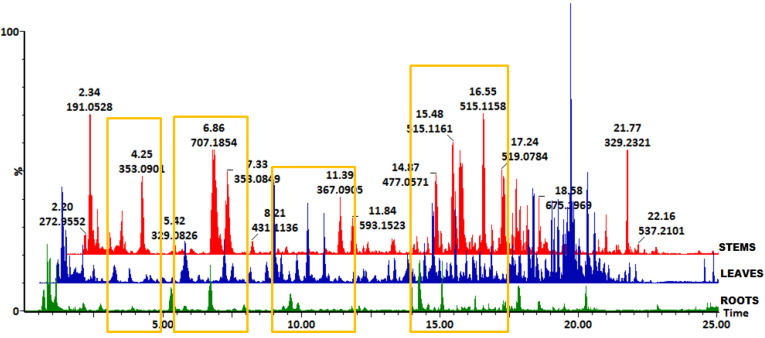
Representative UHPLC-QTOF-MS base peak intensity (BPI) chromatograms showing the separation of secondary metabolites in extracts of *B. pilosa* roots (green), leaves (blue), and stems (red). The yellow rectangles indicate the chromatographic regions where hydroxycinnamic acid derivatives are present across the three tissue types with some visible differences in intensities.

In this study, the distribution profile of HCA derivatives in tissues (leaves, stems, and roots), callus and cell suspensions derived from stems and leaf tissues were studied with the aid of a high-throughput analytical method, UHPLC-QTOF-MS (Figure 1 and Figure S1A,B respectively). Structural elucidation and putative annotations were achieved through an ISCID method [34,35]. The HCA derivatives were shown to be a prominent group of metabolites in methanol extracts of *B. pilosa* tissues (Figure 1) and extracts from stem- and leaf-derived callus and cell suspensions (Figure S1A,B), shown in base peak intensity (BPI) chromatograms and indicated with yellow rectangles. Furthermore, the BPI chromatograms indicated differences in intensities of the HCA derivatives as shown in Figure 1. Within a specific plant species, the distribution of secondary metabolites is expected to vary not only as a function of developmental stage, but also among plant tissues, hence some tissue-specific metabolite variations are observed [39].

### 2.2. Multivariate Data Analysis to Reveal Tissue-Specific and Cell Line-Specific Differences Within Tissues and Cell Cultures of *B. pilosa*

**Figure 2:**
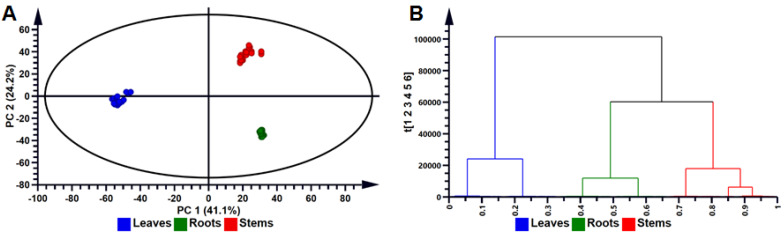
Multivariate data analysis of compositional differences in hydromethanolic extracts of *Bidens pilosa* tissues types. (**A**) A PCA scores scatterplot of the *Pareto*-scaled data set obtained from LC-MS experiments. The six-component model (with PC 1 and PC 2 explaining 65.3% of the variation) indicate the general clustering within the datasets of *B. pilosa* tissues (leaves (blue), stems (red) and roots (green) samples). The quality parameters of the model are: explained variation/goodness-of-fit R^2^ = 0.890 and the predictive variance Q^2^ = 0.874. The ellipse in the PCA score scatterplot indicates the Hotelling’s T^2^ at 95% confidence interval. (**B**) Hierarchical cluster analysis of the hierarchical structure of the data in dendrogram format. The model computed (using Euclidean distance and Ward’s minimum variance as a dissimilarity and linkage rule, respectively) shows tissue-specific clustering into two major groups, grouping roots and stems tissues together.

To analyze the variability within and between the tissue extracts (Figure 2A), cell callus (Figure S2A) and cell suspensions (Figure S2B), principal component analysis (PCA) was performed for all metabolites within each sample group. PCA is an unsupervised, explorative chemometric tool for the reduction of dimensionality of complex datasets to provide insights into variations and systematic trends among sample groups [40,41]. The computed model (score plot) of the PCA for tissues indicate that 41.1% and 24.3% of the variation were explained by PC1 and PC2, respectively (Figure 2A). Statistical validation was described using R^2^ and Q^2^ which explains the goodness-of-fit of the model and model predictability, respectively. The model computed was acceptable for metabolomic analysis of the phytochemical data as the R^2^ > 0.7 and the Q^2^ > 0.4 [42]. For the computed model, R^2^ = 0.890 and Q^2^ = 0.874 respectively were found to be statistically adequate to make relevant biological interpretations. The PCA model revealed an obvious separation among the three tissue types, as shown in Figure 2A. This indicates that the metabolic constituents and their distribution in leaf, stem and root tissues of *B. pilosa* varied significantly. This observation is also reflected in the variation of intensities of separated metabolites observed in the BPI chromatograms shown in Figure 1. Similarly, PCA scores plots were computed and validated for stem- and leaf-derived callus and cell suspensions as indicated in Figure S2A and Figure S2B respectively.

To determine correlations/similarities among the different tissues, agglomerative hierarchical cluster (HC) analysis models were computed. Each observation is initially treated as an individual cluster; then after processing, groups are merged to indicate group similarities [43]. These were represented by means of a dendrogram shown in Figure 2B which indicates differences in metabolomic profiles respective to the different tissues of *B. pilosa*. The dendrogram indicates that the metabolomic profiles of stems and roots are closely related, contrasting with that of leaf tissues [44]. Dendrograms were also constructed to indicate similarities within different cell cultures of *B. pilosa* as shown in Figure S2C,D.

### 2.3. Metabolite Annotations

**Figure 3:**
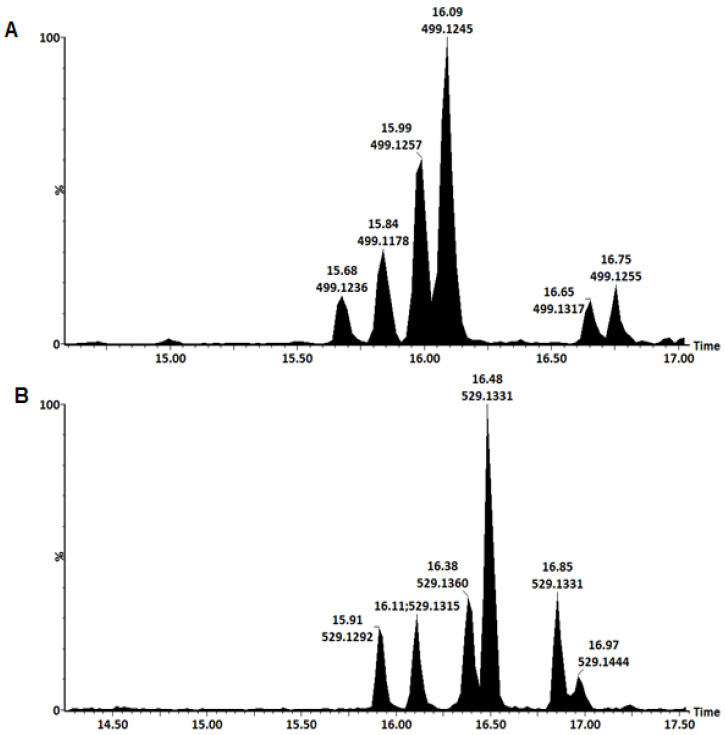
Representative UHPLC-QTOF-MS/MS single ion chromatograms showing the separation of coumaroyl-caffeoylquinic acids (**A**) and feruloyl-caffeoylquinic acids (**B**).

**Figure 4:**
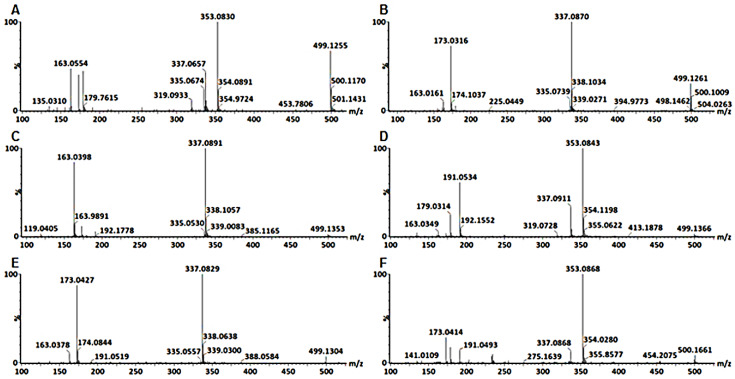
Typical mass spectra of the fragmentation patterns of 3-coumaroyl-4-caffeoylquinic acid (**A**), 3-caffeoyl-4-coumaroylquinic acid (**B**), 3-coumaroyl-5-caffeoylquinic acid (**C**), 3-caffeoyl-5-coumaroylquinic acid (**D**), 4-coumaroyl-5-caffeoylquinic acid (**E**) and 4-caffeoyl-5-coumaroylquinic acid (**F**).

**Figure 5:**
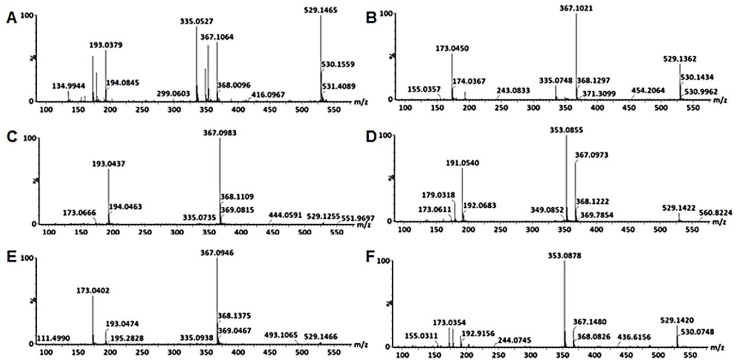
Typical mass spectra of the fragmentation patterns of 3-feruloyl-4-caffeoylquinic acid (**A**), 3-caffeoyl-4-feruloylquinic acid (**B**), 3-feruloyl-5-cafffeoylquinic acid (**C**), 3-caffeoyl-5-feruloylquinic acid (**D**), 4-feruloyl-5-caffeoylquinic acid (**E**), and 4-caffeoyl-5-feruloylquinic acid (**F**).

**Figure 6:**
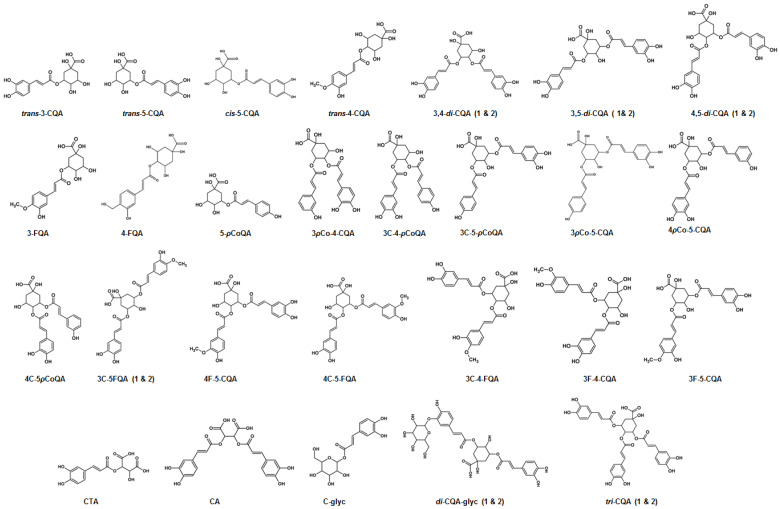
Chemical structures of *mono*-, *di*- and *tri*-substituted hydroxycinnamic acid (HCA) derivatives of quinic acid (QA) and tartaric acid (TA) identified in tissues and two suspension-cultured cell lines of *Bidens pilosa*.

Mass spectral data were obtained in both ESI (+/-) modes, but negative ionization was preferred as the majority of metabolites were found to ionize better in the negative MS mode [31,32] and since the hierarchical scheme proposed by Clifford et al. [31] was also generated using negative ionization. In this study a combined total of 33 CGA derivatives (both *regio*- and geometric isomers) were identified in *B. pilosa* tissues, callus and cell suspensions as listed in Table 1 with corresponding MS data in Figure 3, Figure 4 and Figure 5 and structures presented in Figure 6. CGAs are classically described as a subclass of phenylpropanoids formed when HCAs are esterified to QA, while a wider scope of the definition describes the CGAs as conjugates of many other compounds such as malic acid, succinic acid, fumaric acid, tartaric acid and sugars [27,32,45]. QA has axial hydroxyl groups attached to carbon positions 1 and 3 and equatorial hydroxyl groups attached to carbons 4 and 5 where the HCAs attach to form CGAs [32]. HCAs are naturally synthesized with *trans* configurations but it has been shown that the *cis* configuration can occur as a result of UV radiation [46,47] and under stress conditions [25]. CGAs are chemically diverse and the various *regio*- and geometrical isomers can make discrimination challenging. UHPLC coupled to CID during tandem MS/MS procedures (such as UHPLC-Q-TOF-MS) can partially solve this problem and identify CGAs through the use of fragmentation patterns as outlined in the discussion [34,45,48]. Metabolites were putatively identified to level 2 of the Metabolomics Standards Initiative (MSI) [49].

## 3. Discussion

The number system used below refers to the sequence of presentation in Table 1 where metabolites are listed according to increasing *m*/*z* values.

### 3.1. Characterization of Mono-Acyl Chlorogenic Acids (CGAs)

The hierarchical scheme keys for LC-MS^n^ identification of CGAs [31] was used to assist in the identification of metabolite **(2)** with a molecular ion [M-H]^-^ at *m*/*z* 337, identified as 5-coumaroylquinic acid as it produced a fragment ion at *m*/*z* 191 [QA-H]^-^ showing loss of a coumaroyl moiety (Figure S3). Metabolites **(4-7)** with a precursor ion [M-H]^-^ at *m*/*z* 353, were annotated as caffeoylquinic acids (CQAs). Previous work [34,35,47] was also used as references in putatively identifying these metabolites listed in Table 1. Mass spectrometric data and chromatographic elution order were also considered while determining the *regio*- and geometric isomers of the annotated metabolites. Notably, *cis*-isomers of 4- and 3-CQAs elute before their *trans*-isomers, while the *cis*-isomer of 5-CQA elute after its *trans*-isomer and after the 4-CQA [46]. 4-CQAs (Figure S4B) can be identified by the presence of an intense *m*/*z* 173 [QA-H-H_2_O]^-^ base peak, while 3-CQAs (Figure S4A) are known to produce a base peak ion at *m*/*z* 191 [QA-H]^-^ and a secondary ion at *m*/*z* 179 [CA-H-H_2_O]^-^ at about 50% intensity of the *m*/*z* 191 base peak [31]. Fragmentation of 5-CQAs (Figure 4C) result in the formation of a single base peak product ion at *m*/*z* 191. Hence, based on the elution order and fragmentation patterns, **(4-7)** were identified as *trans*-3-CQA **(4)**, *trans*-5-CQA **(5)**, *trans*-4-CQA **(6)**, and *cis*-5-CQA **(7)** respectively. In tissues, all the *trans*-CQAs were present with the exception of *trans*-3-CQA which was absent in roots. In callus cultures only *trans*-5-CQA was found to be absent. The *mono*-CQAs were observed in cell suspensions derived from both stems and leaves of *B. pilosa*; however, the *cis*-5-CQA was only present in cell suspensions derived from leaf tissue.

A similar approach was followed in the identification two feruloylquinic acid (FQA) isomers **(8** and **9)** which were identified by their precursor ion [M-H]^-^ at *m*/*z* 367 and based on their fragmentation patterns and Rt shown in Table 1. Although differing in intensities, the two FQA *regio*-isomers were identified in all tissues and cell culture systems. As described in the hierarchical scheme keys for LC-MS^n^ identification of CGAs [31], base peaks at *m*/*z* 193 [FA-H]^-^ and *m*/*z* 173 [QA-H-H_2_O]^-^ were used as diagnostic peaks for 3-FQA (Figure S5A) and 4-FQA (Figure S5B) respectively. FQA also produces *m*/*z* 134 [FA-H-CO_2_-CH_3_]^-^. Hence molecules **(6)** and **(7)** were annotated as 3-FQA acid **(6)** and 4-FQA **(7)** respectively.

### 3.2. Characterization of Caffeoylglycoside

HCAs in nature may occur as soluble forms conjugated to organic acids and/sugars [50]. In this study, one caffeoylglycoside **(3)** was identified which had a precursor ion, [M-H]^-^, at *m*/*z* 341 as shown in Table 1. The molecular ion fragmented to give ions at *m*/*z* 179 [CFA-H]^-^ due to loss of a glycosyl residue and an ion at *m*/*z* 135[CFA-CO_2_]^-^ shown in Figure S6 [35]. The caffeoylglycoside was found to be present in all the tissues of *B. pilosa*.

### 3.3. Characterization of Hydroxycinnamoyl-Tartaric Acid Esters

Hydroxycinnamoyl-tartaric acid esters such as the *di*-ester of two caffeic acids to tartaric acid (chicoric acid, CA) and the *mono*-ester of caffeic acid to tartaric acid (caftaric acid, CTA), are biologically active compounds which are shown to have various health benefits and antioxidant properties [51,52]. These HCA derivatives are the main caffeic acid derivatives in *Echinacea purpurea* but have been also identified in leaves of *B. pilosa* and in more than 60 plant genera [53,54]. Caftaric acid (Figure S7A) **(1)** was identified by its parent ion, [M-H]^-^, at *m*/*z* 311 which fragmented to produce ions at *m*/*z* 179 [CFA-H]^-^ due to the loss of a tartaric acid (TA) residue, *m*/*z* 149 [TA-H]^-^ and *m*/*z* 135 [FA-CO_2_]^-^ which resulted from decarboxylation of the caffeic acid residue. Therefore, **(1)** was identified as a hydroxycinnamoyl-tartaric acid ester, CTA (Table 1). Metabolite **(10)** was identified as CA (Figure S7B) by its molecular ion [M-H]^-^, at *m*/*z* 473 which fragmented to give product ions at *m*/*z* 311 [CTA-H]^-^ due to the loss of a second caffeic acid residue. Other daughter ions at *m*/*z* 149 [TA-H]^-^ and *m*/*z* 135 [CFA-CO_2_]^-^ were also observed [29]. Profiling of the tartaric esters revealed that they are only present in the aerial parts of the plants and absent in the roots. This could suggest exclusivity in the biosynthesis of tartaric acid esters, suggesting localized biosynthesis of these esters in the aerial parts of the plant. These esters were also absent in cell cultures of *B. pilosa*.

### 3.4. Characterization of Di-Caffeoylquinic Acids (Di-CQAs)

In this study, six *di*-CQAs shown in Table 1 which produced a molecular ion, [M-H]^-^, at *m*/*z* 515 were identified **(17–22)**. As shown in the hierarchical scheme for LC-MS^n^ identification of CGAs, 3,5-*di*-CQA (**19** and **20)**, Figure S8B, can be differentiated from the other present *regio*-isomers as no base peak of *m*/*z* 173 [QA-H_2_O-H]^-^ was observed which represents the absence of a 4-acyl substitution. To differentiate between 3,4-*di*-CQA (**17** and **18**), Figure S8A and 4,5-*di*-CQA (**21** and **22)**, Figure S8C, an intense ion at *m*/*z* of 335 [CQA-H_2_O-H]^-^ is noteworthy as it represents 3,4-*di*-CQA in an MS^2^ acquisition. Although it may be present in the fragmentation pattern of 4,5-*di*-CQA, the intensity is comparatively lower. Differences observed in the intensities of fragment ions can be ascribed to variances in energy distribution which cause the structurally similar isomers to behave differently under the described MS conditions. On a reverse-phase column the elution order of *di*-CQA *regio*-isomers is expected to be as follows: 3,4-*di*-CQA, 3,5-*di*-CQA, followed by 4,5-*di*-CQA eluting the latest, validating the annotation of *di*-CQAs shown in Table 1 [21,29]. These *di*-CQA were differentially present throughout tissues and cell cultures of *B. pilosa*.

### 3.5. Characterization of Tri-Caffeoylquinic Acids (Tri-CQAs) and Di-Caffeoylquinic Acid Glycosides

Four isobaric peaks with parent ions, [M-H]^-^ at *m*/*z* 677 were observed. Metabolites **(30)** and **(31)** were annotated as *di*-caffeoylquinic acid glycosides (Figure S9B) as these metabolites fragmented to produce ions at *m*/*z* 515 [diCQA-H]^-^ resulting from the loss of a glucosyl residue, *m*/*z* 353 [CQA-H]^-^ resulting from loss of a caffeic acid and glucosyl residue, *m*/*z* 341 [CQA glycoside-H]^-^ which indicated subsequent loss of the quinic acid residue. Other secondary ions observed were at *m*/*z* 179 [Caffeic acid-H]^-^, and at *m*/*z* 173 [Quinic acid-H_2_O-H]^-^ as shown in Table 1 [55].

Metabolites **(32)** and **(33)** were annotated as *tri*-CQAs (Figure S9A) as these produced fragment ions at *m*/*z* 515 [diCQA-H]^-^, *m*/*z* 353 [CQA-H]^-^, *m*/*z* 335 [caffeoylquinic acid-H_2_O-H]^-^, *m*/*z* 191 [QA-H]^-^, *m*/*z* 179 [CFA-H]^-^, and at *m*/*z* 173 [QA-H-H_2_O]^-^. The Rt was also considered as *tri*-CQAs are expected to elute later than 4,5-*di*-CQA as these are more hydrophobic [56]. However, positions of acylation on *tri*-caffeoylquinic acids/*tri*-acylated glycosides were not fully characterized as description of *tri*- and tetra-acylation would require MS^4^ and/or MS^5^ spectra [32]. These *tri*-acylated HCA derivatives were observed to be only present in the leaves of *B. pilosa*.

### 3.6. Characterization of p-Coumaroyl-Caffeoylquinic Acids (pCo-CQAs)

As mentioned earlier, CGAs are a complex group of compounds that may also comprise of mixed *di*-esters. Characterization of *p*-coumaric acid-containing *di*-acyl-CGAs has been previously done for green coffee beans [53]. To the authors’ knowledge, these have never been identified in *B. pilosa*. *p*Co-CQAs were identified by a parent ion [M-H]^-^ at *m*/*z* 499 [22,57]. The elution order of these metabolites was considered to assist in their annotation. Six of these isomers were observed **(11–16)** and they occurred in pairs as shown in Figure 3A and Table 1. These isomers were found to follow an elution order similar to that of *di*-CQAs where 3,4-*di*-esters elute first, followed by the 3,5-*di*-esters and with the 4,5-*di*-esters eluting last. The 20 eV collision energy level (MS^E^) was considered when annotating these metabolites.

The first pair was annotated as 3-*p*Co-4-CQA **(11)** and 3-C-4-*p*CoQA **(12)** shown in Figure 4A,B respectively. 3-ρCo-4-CQA **(11)** fragments produced a base peak ion at *m*/*z* 353 [CQA-H]^-^ and secondary ions at *m*/*z* 337 [*p*CQA-H]^-^, 335 [CQA-H_2_O-H]^-^, *m*/*z* [QA-H]^-^, *m*/*z* 173 [QA-H-H_2_O]^-^, 163 [*p*CoA-H]^-^. The presence of a peak at *m*/*z* 173 was indicative of acylation at C4 of the QA, the peak at *m*/*z* 335 indicated a dehydrated caffeoylquinic acid and its ratio of approximately 30% to the base peak was characteristic of a 3,4-*di*-chlorogenic acid. An intense product ion at *m*/*z* 163 is characteristic of a coumaric residue at C3 of the QA. The 3-C-4-*p*CoQA **(12)** fragmented to give a base peak *m*/*z* 337, which is indicative of a loss of a caffeoyl residue and secondary peak at *m*/*z* 173 which indicated that the coumaroyl residue was acylated at C4 of the QA.

The next pair of isomers were identified as 3,5-*di*-esters due to their lack of a product ion at *m*/*z* 173. Figure 4C indicates the fragmentation pattern of a metabolite annotated as 3-*p*Co-5-CQA **(13)** with a base peak at *m*/*z* 337 indicating that the caffeoyl residue was extensively lost and which suggests that the caffeoyl residue is attached at position C5 of QA. According to [31] the acylation at position C5 is the easiest to remove followed by that at position C3, whilst the one at C4 is the hardest to remove. A product ion at *m*/*z* 163 was also observed which indicated that the coumaroyl residue was attached at position C3, hence this metabolite was annotated as 3-*p*Co-5-CQAs **(13)**. The other isomer of this pair was annotated as 3-C-5-*p*CoQA **(14)**. Its fragmentation pattern is indicated in Figure 4D, showing a base peak at *m*/*z* 353 and secondary ions of *m*/*z* 337, 191 and 179. The caffeoyl residue was assigned position C3 as the secondary ions *m*/*z* 191 and 179 showed behavior analogous to that of 3-CQA were a base peak at *m*/*z* 191 is observed and an ion at *m*/*z* 179 is present at 50% intensity compared to the *m*/*z* 191.

The last two pairs were annotated as 4,5-*di*-esters and are indicated in Figure 4E and Table 1. The isomer that eluted first in this pair was annotated as 4-coumaroyl-5-caffeoylquinic acid **(15)** and the fragments observed were a base peak at *m*/*z* 337 and secondary ions at *m*/*z* 173 and *m*/*z* 163, also shown in Figure 4E. Absence of the *m*/*z* 353 suggested that the caffeoyl residue was more extensively lost and most likely attached at position C5 of QA. The base peak at *m*/*z* 337 gave a fragment at *m*/*z* 173, suggesting that the coumaroyl residue was attached to position C4 of QA. The last isomer was annotated as 4-C-5-*p*CoQA **(16)**. The fragmentation pattern of this metabolite is shown in Figure 4F, with a base peak at *m*/*z* 353 and secondary ions at *m*/*z* 337, 191, 179 and 173. All these isomers were present in both stem and leaf cell suspensions but absent in callus cultures. Five of these isomers were observed in leaf tissue of *B. pilosa* but were undetected in the stem and root tissues.

### 3.7. Characterization of Feruloyl-Caffeoylquinic Acids (F-CQAs)

The F-CQAs **(23–29)** were identified by their parent ion at *m*/*z* of 529 and the chemical structures are illustrated in Figure 6 [22,31,57]. Chromatographically, six isomers were observed which eluted in pairs shown in Figure 3B. The first two isomers were identified as 3-F-4-CQA **(23)** and 3-C-4-FQA **(24)**. A typical fragmentation pattern of 3-F-4-CQA **(23)** shown in Figure 5A indicated fragment ions at *m*/*z* 367 [FQA-H]^-^, *m*/*z* 353 [CQA-H]^-^, *m*/*z* 335 [CQA-H_2_O-H]^-^, *m*/*z* 193 [FA-H]^-^, *m*/*z* 179 [CA-H-H_2_O]^-^, *m*/*z* 173 [QA-H-H_2_O]^-^ and at *m*/*z* 134 [FA-H-CO_2_-CH_3_]. An intense product ion at *m*/*z* 335 indicated that this isomer was a CGA with the caffeoyl residue attached to position C4 of QA. The presence of an intense *m*/*z* 193 ion indicated that the feruloyl residue was attached at position C3 of QA. Figure 5B shows the fragmentation of 3-C-4-FQA **(24)** which gave a base peak of *m*/*z* 367 and secondary ion at *m*/*z* 173, indicating that the feruloyl residue is attached at position C4 of QA.

The next two isomers were annotated as 3,5-*di*-esters as they lacked a fragment ion of *m*/*z* 173 which indicated no acylation at position C4 of QA. These were annotated as 3-F-5-CQA **(25)** and 3-C-5-FQA-1 **(26)**. The fragmentation pattern of 3-F-5-CQA **(25** – Figure 5C) shows a base peak at *m*/*z* 367 which indicates extensive loss of the caffeoyl residue. This suggests acylation with a caffeoyl residue at position C5, while the intense secondary ion at *m*/*z* 193 indicates that the feruloyl residue was attached to position 3. Figure 5D shows the fragmentation pattern of 3-C-5-FQA **(26)** and fragments observed were *m*/*z* 353, 337, 191 and 179. The caffeoyl residue was assigned to position C3 as ions *m*/*z* 191 and 179 showed behavior similar to that of 3-CQA where a base peak at *m*/*z* 191 is observed and an *m*/*z* at 179 is present at 50% intensity compared to the *m*/*z* 191 ions. The feruloyl moiety was thus assigned to position C5 of QA.

The last two isomers were annotated as 4-F-5-CQA **(27)** and 4-C-5-FQA **(28)**. The first eluting isomer showed a base peak *m*/*z* 367 and secondary ions *m*/*z* 193 and an intense *m*/*z* 173 shown in Figure 5E, hence the feruloyl residue was attached at position C4. Absence of *m*/*z* 353 indicated acylation of the caffeoyl residues at position C5 of QA. The fragmentation pattern of 4-C-5-FQA **(22)** is shown in Figure 5F. A later eluting F-CQA was also identified in stems and leaf tissues. This metabolite was identified as 3-C-5-FQA-2 **(29)** as it had similar fragmentation ions to **(26)**.

### 3.8. Distribution of HCA Derivatives in Tissues and Cell Cultures of *B. pilosa*

Differential distribution of HCA derivatives were observed amongst the various tissue types of *B. pilosa* as indicated in Table 1. Most annotated HCA-derivatives (specifically quinic acid esters) were found to be mostly distributed in stem and leaf tissues as opposed to the roots. As described in [58], CGAs were found to be distributed mainly in chlorenchyma cells and appeared to be associated with chloroplast and were implicated to confer protection to chloroplasts against light. These metabolites were also found to be localized in the vascular bundles and this could suggest that they are transported throughout plant organs. From the observations of the distribution of CGAs in *B. pilosa* tissues, this suggests that the synthesis of these metabolites could be localized in the aerial parts of the plant and possibly translocated to other plant organs. Furthermore, this suggests that in *B. pilosa*, the hydroxycinnamoyl-CoA/quinate hydroxycinnamoyl transferase (HQT), an enzyme responsible for the biosynthesis of quinic acid esters [59] could be localized in or near the chloroplast, hence accumulation of HCA-derivatives was higher in the aerial parts of the plant.

In contrast, the tartaric acid esters were only to be distributed in the aerial parts of the plant and mostly in leaves whilst absent in root tissues. This corresponded to the analysis of different tissues of *E. purpurea* where CA was found to be present more in the apical parts of that plant [60]. Absence of the tartaric acid esters (CA and CTA) in the root tissues of *B. pilosa* could suggest a few possibilities that would require further investigations. Metabolite distribution patterns may differ, due to differences in expression of genes and localization of enzymes responsible for their biosynthesis [61]. The hydroxycinnamoyl-CoA/tartaric acid hydroxycinnamoyl transferase (HTT), an enzyme responsible for the biosynthesis of the tartaric esters [62] could be localized in the aerial parts of the plant considering the apparent complete absence of these esters in roots, therefore suggesting its localization in the chloroplast. However, further proteomics studies are required to validate the localization of HTT in the chloroplast and measurements of posttranscriptional regulation would need to be conducted to connect the distribution of these esters to localization of HTT. Deducing from the observations, HCA derivatives were generally abundant in the aerial parts of the plant; this could provide a chemical basis for the distinct usage of different tissues of *B. pilosa*, to maximally harness its bioactivities.

Plant cell cultures have been shown to be an attractive approach for the controlled production of bioactive natural products like phenylpropanoids, compared to the use of wild plants [11]. In cell suspensions of *B. pilosa*, 23 HCA-derivatives were identified while only 14 of these were identified in the callus cultures as presented in Table 1. Although plant cell culture is a promising alternative for metabolite production and provides numerous advantages, a significant limitation of using cell culture is that undifferentiated cells may accumulate secondary metabolites to a lesser extent compared to the parent plant [63,64]. As observed in this study, fewer HCA-derivatives were accumulated in cell cultures compared to plant tissues where 30 HCA-derivatives were identified. However, various strategies can be employed to improve yield of secondary metabolites in plant cell cultures such as optimizing cultural conditions (medium modifications) [65], precursor feeding [66], immobilization techniques [11], elicitation [67], and screening for high-producing cell lines [14]. As mentioned above, fewer HCA-derivatives were identified in cultured callus cells compared to cell suspensions. This could be because callus is maintained on semi-solid media while cell suspensions are maintained in liquid medium which is agitated to enhance oxygenation, promote better growth and transfer of nutrients [68,69].

Cell suspensions of *B. pilosa* were shown to be better starting material for in vitro cultivation for production of HCA-derivatives such as CGAs. Cultured cells of *B. pilosa* were noted to possess some form of a genetic memory and totipotency for the biosynthesis of some CGAs, similar to that of the parent plant. This observation may in future provide possibilities for bioreactor-based large-scale production of these biologically important secondary metabolites. Cultured cells are comparable to undifferentiated meristematic cells and lack chloroplasts. This could explain the apparent lack of tartaric acid esters in *B. pilosa* cultures [70]. These esters were hypothesized to be exclusively biosynthesized by the enzyme HTT as mentioned above which possibly could be localized in the chloroplasts of *B. pilosa*. Although making photosynthetic cell cultures with functional chloroplasts has been deemed difficult and time-consuming, recent advances have been made where medium modification in *Arabidopsis thaliana* cultures resulted in chloroplast formation [71].

## 4. Materials and Methods

### 4.1. Plant Cultivation Tissues and Undifferentiated Cells

*B. pilosa* seeds were collected from matured plants in the wild (Venda area of South Africa) and air-dried at room temperature. The seeds were cold shocked at 4 °C for 48 h; this was performed as a way of cold-wet stratification to break seed dormancy in summer perennials [72]. The seeds were then sown in Culterra germination mix (Culterra, Muldersdrift, South Africa, http://culterra.co.za). Germinated plants were then grown under greenhouse conditions at 28 °C for a period of two months. The plants were watered twice a week and fertilized once every two weeks with a fertilizer containing 90 mg/L mono-potassium phosphate, 150 mg/L Soluptase, 20 mg/L Microples, 40 µL/L Kelp-P-Max, 650 mg/L CaNO3, 400 mg/L KNO3, 300 mg/L MgSO4, and 90 mg/L mono-ammonium phosphate. Plants stems, roots and leaves were harvested and immediately shock-frozen in liquid nitrogen to quench all metabolic reactions [73,74,75]. The frozen plant tissues were stored at −80 °C, pending metabolite extractions.

### 4.2. Callus Initiation and Cell Suspension Cultures

*B. pilosa* callus cultures were established from leaf and stem explants on Murashige and Skoog medium with Murashige and Skoog vitamins containing 100 mg/L myo-inositol, 1 g/L hydrolyzed casein and 30 g/L sucrose with agar. The medium contained growth regulators, 0.45 mg/L 2,4-dichlorophenoxyacetic acid (2,4-D) and 1 mg/L 6-benzylaminopurine (BAP) at pH 5.8. Friable callus was sub-cultured into the medium with 0.45 mg/L 2,4-D and 1.0 mg/L BAP and grown in Erlenmeyer flasks on an orbital shaker at 120 rpm at room temperature with a light/dark cycle of 12 h/12 h and maintained at a low light intensity of 30 µmol/m^2^/s.

### 4.3. Metabolite Extraction

Two grams (2 g) of the samples; cell suspensions (harvested using filter paper (70 mm) on a vacuum filtration system), callus and plant tissues (crushed frozen with liquid nitrogen using a mortar and pestle) were homogenized at 5100 rpm in 20 mL (1:10 m/v) of 80% methanol (Romil SpS, Cambridge, UK). Samples were sonicated for 30 min at 30 °C and 100% intensity in a sonicator bath (Branson CPX, Fischer Scientific, Waltham, MA, USA). The crude extracts were centrifuged at 5100 rpm in a benchtop centrifuge (Beckman Coulter, Midrand, South Africa), for 15 min and supernatants were evaporated under vacuum using a rotary evaporator (Heidolph Instruments, Schwabach, Germany), at 55 °C to ~1 mL of samples. Samples were transferred to 2 mL Eppendorf tubes and dried to completion overnight in a dry bath at 55 °C, reconstituted with 500 µL of 50% methanol and sonicated for 30 min at 30 °C followed by filtration using 0.22 µm nylon filters into HPLC glass vials with 500 µL inserts. Samples were stored at 4 °C until future analysis. To ensure experimental reproducibility, eight independent biological replicates were prepared, and three instrumental technical replicates were analyzed.

### 4.4. Ultra High-Performance Liquid Chromatography-Quadrupole Time-of-Flight Mass Spectrometry (UHPLC-QTOF-MS/MS)

Extracts were analyzed on an ultrahigh-performance liquid chromatography-quadrupole time-of-flight MS instrument (UHPLC-QTOF SYNAPT G1 system, Waters Corporation, Manchester, UK) fitted with an Acquity HSS T3 C18 column (150 mm × 2.1 mm with particle size of 1.7 μm) (Waters, Milford, MA, USA). An injection volume of three µL was used and a binary solvent system was used consisting of solvent A: 0.1% formic acid in Milli-Q water (both HPLC grade, Merck, Darmstadt, Germany) and solvent B: acetonitrile (UHPLC grade, Romil SpS, Cambridge, UK) with 0.1% formic acid. A binary solvent gradient (with solvent A and B) with a flow rate of 0.4 mL/min was used to separate analytes over 30 min. The separation conditions were: 2% B over 0.0–1.0 min, 2%–60% B over 2.0–24 min, 60%–95% B over 24–25 min, from 25–27 min the conditions were maintained at 95% B and the column was washed with 95%–2% B over 27–28 min. The column was allowed to re-equilibrate with 5% B over a 2 min isocratic wash. The chromatographic effluents were further analyzed utilizing the SYNAPT G1 Q-TOF high definition mass spectrometer (Waters Corporation, Manchester, UK). Separate injections (using the same chromatographic settings and conditions) were performed for positive and negative electrospray ionization (ESI) modes. The MS conditions were set as follows: capillary voltage of 2.5 kV, sampling cone voltage of 30 V, extraction cone of 4.0 V, source temperature of 120 °C, cone gas flow of 50.0 (L/h), desolvation gas flow of 550 (L/h), *m*/*z* range of 100–1000, scan time of 0.2 sec, interscan delay of 0.02 sec, mode: centroid. Leucine encephalin was used as a reference calibrant (50 pg mL^−1^, [M + H]^+^ = 556.2766 and [M – H]^−^ = 554.2615), continuously sampled every 15 sec, producing an average intensity of 350 counts scan^−1^ in centroid mode, with typical mass accuracies between 3–5 mDa. For downstream structural elucidation, the MS analysis was set to result in both unfragmented and fragmentation experiments (MS^E^) by ramping the collision energy from 15 to 60 eV in a series of for fragmentation experiments. For untargeted analysis these conditions were kept constant throughout.

For the targeted approach, linked to ISCID, conditions were varied. Fragmentation patterns of pre-selected HCA derivatives with parent ions [M-H]^-^ at *m*/*z* 677 (tricaffeoylquinic acids), *m*/*z* 529 (feruloycaffeoylquinic acids), *m*/*z* 499 (coumaroylquinic acids), *m*/*z* 515 (dicaffeoylquinic acid), *m*/*z* 367 (feruloylquinic acid), *m*/*z* 353 (caffeoylquinic acids), *m*/*z* 337 (coumaroylquinic acid) were chosen for MS^2^ analysis. Fragmentation was achieved by the ISCID method as explained [34,35,36,37,38] where the collision energy (10–40 eV) and the cone voltage (10–100 V) were optimized to produce fragment ions of chlorogenic acids at *m*/*z* 191 [quinic acid-H]^−^, *m*/*z* 179 [caffeic acid-H-H_2_O] ^−^, *m*/*z* 173 [quinic acid-H-H_2_O]^-^ and *m*/*z* 135 [caffeic acid-H-CO_2_]^-^ [34,35].

### 4.5. Data Processing, Multivariate Data Analysis (MVDA), and Metabolite Annotations

Prior to MVDA, UHPLC-QTOF-MS raw data was processed using MassLynx XS™ software’s MarkerLynx application (Waters, Manchester, UK). The MarkerLynx application performs accurate peak detection and alignment using the patented *ApexTrack* algorithm [41]. The following parameters were used: Retention time (Rt) range of 0.50–22 min with a Rt window of 0.2 min, mass range of 100–1000 Da and the mass tolerance as 0.05 Da. After the peaks were detected the corresponding ions (consisting mostly of the pseudomolecular ion peaks before fragmentation) were analyzed (maximum intensity, Rt and *m*/*z*) and recorded for all the samples. Data normalization was done by using total ion intensities of each defined peak. Prior to calculating intensities, the software performs a patented modified Savitzky-Golay smoothing and integration. The data matrix obtained from MassLynx was exported into SIMCA-15.0 software (Umetrics Corporation, Umea, Sweden) for statistical modeling. Statistical models computed were principal component analysis (PCA) and hierarchical cluster (HC) analysis which are both unsupervised models that show trends, clusters and similarities between samples. Agglomerative HC models were computed using Ward’s linkage method (incremental sum of squares method) that considers between- and within-cluster distances when forming clusters, and the tree was sorted based on size [37]. Unless stated otherwise, *Pareto* scaling was applied for all computed models to reduce the relative importance (masking effects) of large values from abundant metabolites, but partially preserve data structure [44,76,77]. Metabolite annotation was executed based on mass spectral information from MS^E^ and/or MS^2^ experiments, accurate mass information, elemental composition calculations and searches in various databases such as ChemSpider and Dictionary of Natural Products (DNP, dnp.chemnetbase.com). Fragmentation patterns were also compared to available literature such as the hierarchical scheme keys for LC-MS^n^ identification of CGAs [31]. Metabolites were putatively identified to level 2 of the Metabolomics Standards Initiative (MSI) [49]. The surrogate standard approach, through comparison with already analyzed plant extracts, was also followed to validate the identity of metabolites of which authentic standard are not available [78].

## 5. Conclusions

In this study tissues, callus and cell suspensions of *B. pilosa* were shown to contain diverse HCA derivatives of quinic acid which substantiates the reported health benefits of the plant as an alternative food source and, moreover, the use of cell culture for production of biologically important secondary metabolites. These HCA derivatives were shown to have differential distribution across tissues and cell cultures. Given this point, undifferentiated cells of *B. pilosa* indicated cell line-specific differences in distribution of HCA derivatives as a result of their inherent metabolite memory. The protocol outlined in this study offers possibilities for sustainable production of biochemically important phenolic compounds in cell suspension cultures of *B. pilosa* as reported for the first time in this study. Although the CGAs identified in this study are structurally diverse in terms of their geometric and *regio*-isomerism, the applied UHPLC-QTOF-MS/MS in-source collision-induced dissociation method assisted in annotating and differentiating between the CGA isomers found the cellular extracts. Minor differences in the fragmentation patterns gave characteristic diagnostic peaks that can be used to efficiently elucidate the *regio*-isomers of the HCA derivatives. Fragmentation patterns similar to those described in the hierarchical scheme keys for LC-MS^n^ identification of CGAs [31] were obtained by use of in-source collision-induced dissociation. This method (ISCID) provides an analytical avenue that allows for efficient discrimination of CGA *regio*-isomers unaccompanied by the use of advanced MS technologies. In addition to the CGAs, HCAs esterified to tartaric acid and sugars are also reported in tissues of *B. pilosa* which indicates diversity in metabolite composition of this plant.

## Figures and Tables

**Table 1:** Characterization of chlorogenic acids (CGAs) consisting of hydroxycinnamic acid (HCA) derivatives of quinic acid (QA) and tartaric acid from tissues (L-leaves, S-Stems and R-Roots) and from two callus cell lines (C-L and C-S) and two cell suspension lines (S-L and S-S) of *Bidens pilosa*.

No	*m*/*z*	Mass Error (mDa)	Rt (min)	Fragment ions	Molecular Formula	Metabolite	Abbreviation	L	S	R	C-l	C-s	S-l	S-s
1	311.0392	8.8	4.51	179, 149, 135	C_13_H_12_O_9_	Caftaric acid	CTA	•	•					
2	337.0822	5.4	9.45	191	C_16_H_18_O_8_	5-Coumaroylquinic acid	5-*ρ*CoQA	•	•	•			•	•
3	341.0829	2.4	7.33	179, 135	C_15_H_18_O_9_	Caffeoylglycoside	CFA-glyc	•	•	•				
4	353.084	4.9	3.31	191, 179, 135	C_16_H_18_O_9_	*trans*-3-Caffeoylquinic acid	*trans*-3-CQA	•	•		•	•	•	•
5	353.0821	5.4	6.39	191	C_16_H_18_O_9_	*trans*-5-Caffeoylquinic acid	*trans*-5-CQA						•	•
6	353.0835	5.7	7.12	191, 179, 173, 135	C_16_H_18_O_9_	*trans*-4-Caffeoylquinic acid	*trans*-4-CQA	•	•	•	•	•	•	•
7	353.0884	2.6	9.16	191	C_16_H_18_O_9_	*cis*-5-Caffeoylquinic acid	*cis*-5-CQA	•	•	•	•	•	•	
8	367.1003	5.5	6.52	193	C_17_H_20_O_9_	3-Feruloylqunic acid	3-FQA	•	•	•	•	•	•	•
9	367.0986	3.7	10.91	191, 173	C_17_H_20_O_9_	4-Feruloylquinic acid	4-FQA				•	•	•	•
10	473.0673	2.7	14.89	311, 179, 149, 135	C_22_H_18_O_9_	Chicoric acid	CA	•						
11	499.1211	1.3	15.68	353, 337, 335, 191, 173, 163	C_25_H_24_O_11_	3-Coumaroyl-4-caffeoylquinic acid	3*ρ*Co-4-CQA	•					•	•
12	499.1183	4.5	15.84	337, 335, 173, 164	C_25_H_24_O_11_	3-Caffeoyl-4-coumaroylquinic acid	3C-4-*ρ*CoQA	•					•	•
13	499.1217	0.6	15.99	337, 163	C_25_H_24_O_11_	3-Coumaroyl-5-caffeoylquinic acid	3*ρ*Co-5-CQA						•	•
14	499.12312	1.0	16.09	353, 337,191, 179	C_25_H_24_O_11_	3-Caffeoyl-5-coumaroylquinic acid	3C-5-*ρ*CoQA	•					•	•
15	499.1352	4.2	16.65	337, 173, 163	C_25_H_24_O_11_	4-Coumaroyl-5-caffeoylquinic acid	4*ρ*Co-5-CQA	•					•	•
16	499.1227	1.2	16.75	353, 337,191, 179, 173	C_25_H_24_O_11_	4-Caffeoyl-5-coumaroylquinic acid	4C-5-*ρ*CoQA	•					•	•
17	515.1182	1.0	14.65	353, 335, 191, 179, 135	C_25_H_24_O_12_	3,4-*di*-Caffeoylquinic acid	3,4-*di*-CQA-1	•	•	•	•	•	•	•
18	515.1163	1.7	14.69	353, 335, 191, 179, 173, 135	C_25_H_24_O_12_	3,4-*di*-Caffeoylquinic acid	3,4-*di*-CQA-2	•						
19	515.1210	6.2	14.93	353, 191, 179, 135	C_25_H_24_O_12_	3,5-*di*-Caffeoylquinic acid	3,5-*di*-CQA-1	•	•	•	•	•	•	•
20	515.1170	2.1	15.03	353, 191, 179, 135	C_25_H_24_O_12_	3,5-*di*-Caffeoylqiunic acid	3,5-*di*-CQA-2	•						
21	515.1292	2.2	15.67	353, 335, 191, 179, 173, 135	C_25_H_24_O_12_	4,5-*di*-Caffeoylquinic acid	4,5-*di*-CQA-1	•	•	•	•	•	•	•
22	515.1122	2.0	16.89	353, 191, 179, 173	C_25_H_24_O_12_	4,5-*di*-Caffeoylquinic acid	4,5-*di*-CQA-2	•						
23	529.1315	1.2	15.92	367, 353, 335, 193, 179, 173, 134	C_26_H_26_O_12_	3-Feruloyl-4-caffeoylquinic acid	3F-4-CQA	•	•		•	•	•	•
24	529.1381	1.9	16.11	367, 335, 193, 173	C_26_H_26_O_12_	3-Caffeoyl-4-feruloylquinic acid	3C-4-FQA	•	•		•	•	•	•
25	529.1296	3.5	16.37	367, 193, 134	C_26_H_26_O_12_	3-Feruloyl-5-cafffeoylquinic acid	3F-5-CQA	•	•		•	•	•	•
26	529.1422	0.8	16.49	367, 353, 191, 179	C_26_H_26_O_12_	3-Caffeoyl-5-feruloylquinic acid	3C-5-FQA-1	•	•		•	•	•	•
27	529.1463	1.2	16.86	367, 193, 173	C_26_H_26_O_12_	4-Feruloyl-5-caffeoylquinic acid	4F-5-CQA	•	•		•	•	•	•
28	529.142	4.6	16.97	367, 353, 191, 179, 173, 135	C_26_H_26_O_12_	4-Caffeoyl-5-feruloylquinic acid	4C-5-FQA	•	•		•	•	•	•
29	529.1395	7.5	17.09	353, 191, 179	C_26_H_26_O_12_	3-Caffeoyl-5-feruloylquinic acid	3C-5FQA-2	•	•					
30	677.14	2.3	12.99	515, 353, 341, 353, 179, 173	C_31_H_33_O_17_	*di*-Caffeoylquinic acid glycoside	*di*-CQA-glc-1		•	•				
31	677.16	0.8	14.07	515, 353, 341	C_31_H_33_O_17_	*di*-Caffeoylquinic acid glycoside	*di*-CQA-glc-2		•	•				
32	677.1436	0.3	17.76	515, 353, 335, 191, 179, 173	C_34_H_30_O_15_	*tri*-Caffeoylquinic acid	*tri*-CQA-1		•	•				
33	677.15	3.3	18.21	515, 353,179, 173	C_34_H_30_O_15_	*tri*-Caffeoylquinic acid	*tri*-CQA-2		•	•				

* Shaded squares indicates presence of metabolites.

## Data Availability

The study design information, LC-MS data, data processing and analyses are reported on and incorporated into the main text.

## Supplementary Materials

The following are available online at https://www.mdpi.com/2218-1989/10/5/178/s1. Figure S1: Representative UHPLC-QTOF-MS base peak intensity (BPI) chromatograms showing the separation of secondary metabolite in methanol extracts of callus cultures (A) and cell suspension cultures (B) of *B. pilosa* initiated from leaves (blue) and stems (red) explant materials. Figure S2: (A and C)—Principal component analysis (PCA) score scatterplots indicating tissue-specific differences within callus cultures (A) and cell suspension cultures (C) of *B. pilosa* tissue initiated from leaves (blue) and stems (red) explant material. (B and D)—Hierarchical cluster analysis dendrograms indicating tissue-specific grouping of callus cultures (B) and cell suspension cultures (D) initiated from leaves (blue) and stems (red) explant material. Figure S3: A typical mass spectrum of the fragmentation pattern of 5-coumaroylquinic acid. Figure S4: Typical mass spectra of the fragmentation patterns of 3-caffeoylquinic acid (A), 4-caffeoyquinic acid (B) and 5-caffeoylquinic acid (C). Figure S5: Typical mass spectra of the fragmentation patterns of 3-feruloylquinic acid (A) and 4-feruloylquinic acid (B). Figure S6: A typical mass spectrum of the fragmentation patterns of caffeoylglycoside. Figure S7: Typical mass spectra of the fragmentation patterns of caftaric acid (A) and chicoric acid (B). Figure S8: Typical mass spectra of the fragmentation patterns of 3,4-*di*-caffeoylquinic acid (A), 3,5-*di*-caffeoylquinic acid (B) and 4,5-*di*-caffeoylquinic acid (C). Figure S9: Typical mass spectra of the fragmentation patterns of *tri*-caffeoylquinic acid (A) and *di*-caffeoylquinic acid glycoside (B).
