# Supplementary material for: Profiling of Chlorogenic Acids from Bidens pilosa and Differentiation of Closely Related Positional Isomers with the Aid of UHPLC-QTOF-MS/MS-Based In-Source Collision-Induced Dissociation
Source: Metabolites. 2020 Apr 29;10(5):178. doi: 10.3390/metabo10050178 (PMC7281500; doi:10.3390/metabo10050178)
Supplement: Supplementary file 1 [file metabolites-10-00178-s001.pdf]

Supplementary file

Profiling of chlorogenic acids from *Bidens pilosa* and differentiation of closely related positional isomers with the aid of UHPLC-qTOF-MS/MS-based in-source collision-induced dissociation

Anza-Tshilidzi Ramabulana [1], Paul Steenkamp [1], Ntakadzeni Madala [1,2,*] and Ian Dubery [1,*]

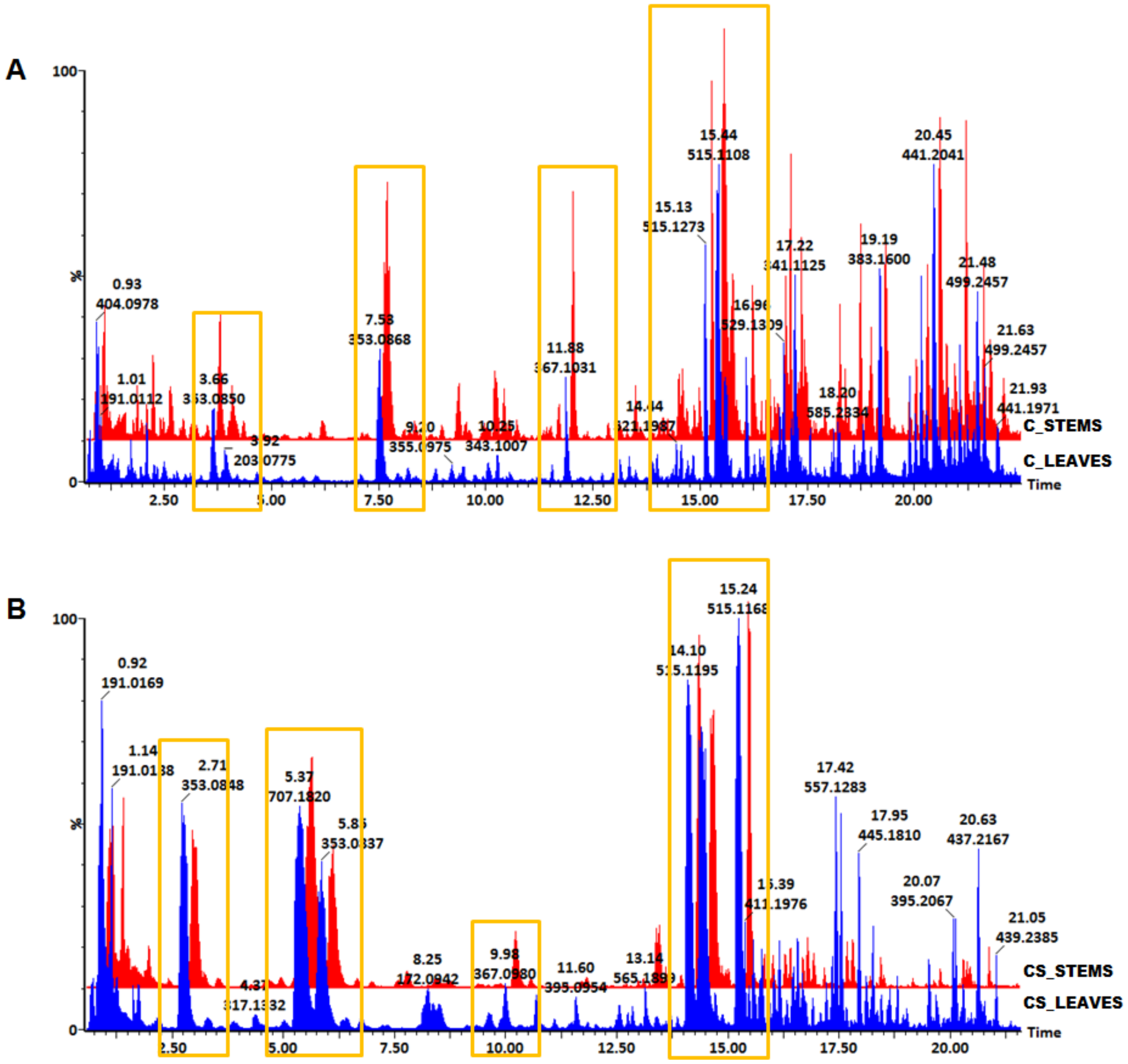

Figure S1: **(A)** Representative UHPLC-QTOF-MS base peak intensity (BPI) chromatograms showing the separation of secondary metabolite in methanol extracts of callus cultures of *B. pilosa* initiated from leaves (blue) and stems (red) explant materials. **(B)** Representative BPI chromatograms indicating metabolites in methanol extracts of cell suspension cultures of *B. pilosa* initiated from friable callus obtained from leaves and stems as explant materials. Hydroxycinnamic acids derivatives that are present across the cell cultures are indicated with yellow rectangles.

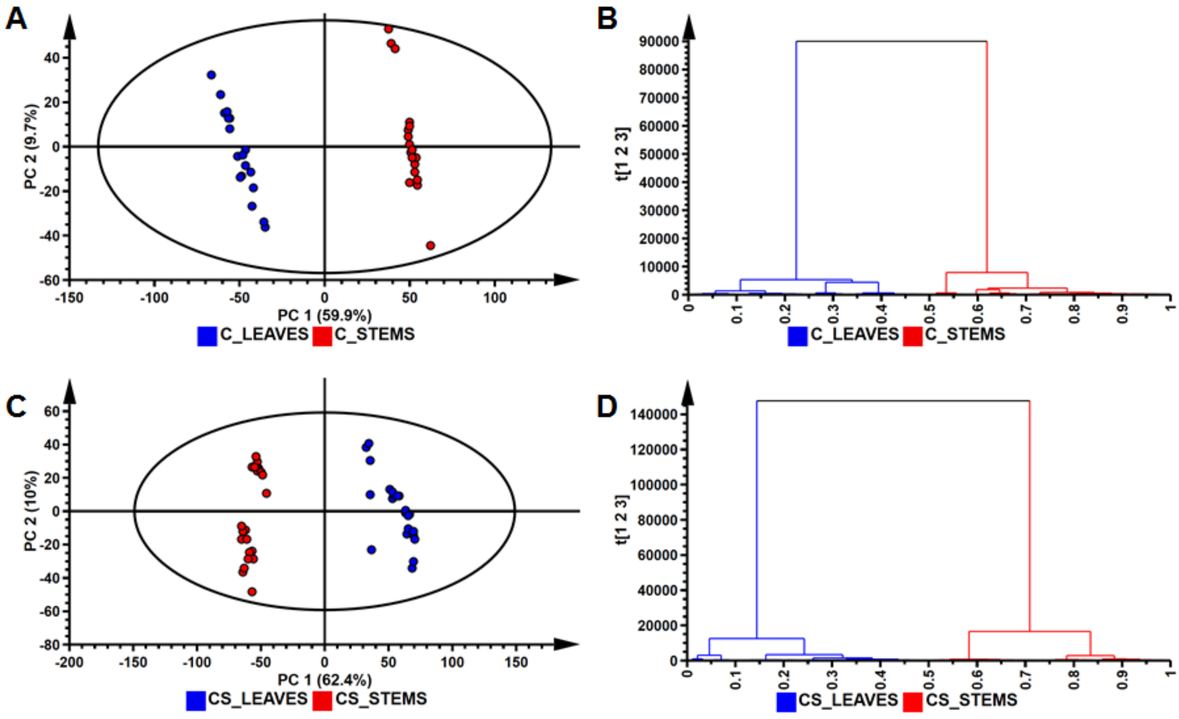

Figure S2: (A): Principal component analysis (PCA) score scatterplots indicating tissue-specific differences within callus cultures of *B. pilosa* tissue initiated from leaves (blue) and stems (red) explant material. The model obtained was a three-component model and explains 69.87% of the variation. The quality parameters of the model were explained variation/ goodness-of-fit R^2= 0.631 and the predictive variance Q^2=0.558. The ellipse in the PCA score scatterplot indicates the Hotelling's T^2 with a 95% confidence interval. **(B):** A hierarchical cluster analysis (HCA) dendrogram showing that the extracts from stem and leaves callus cluster into different groups**. (C):** PCA scores plot of cell suspensions (initiated from callus culture of *B. pilosa* leaves and stems) data set indicating different clustering patterns. The model obtained was a three-component model and explains 72.4% of the variation. R^2= 0.785 and Q^2= 0.703 reported on the goodness-of-fit and the predictive variance of the model. The ellipse in the PCA score scatterplot indicates the Hotelling's T^2 with a 95% confidence interval. **(D):** HCA indicates differences between cell suspensions derived from leaves (CS_Leaves) and stems (CS_Stems), forming the two major nodes.

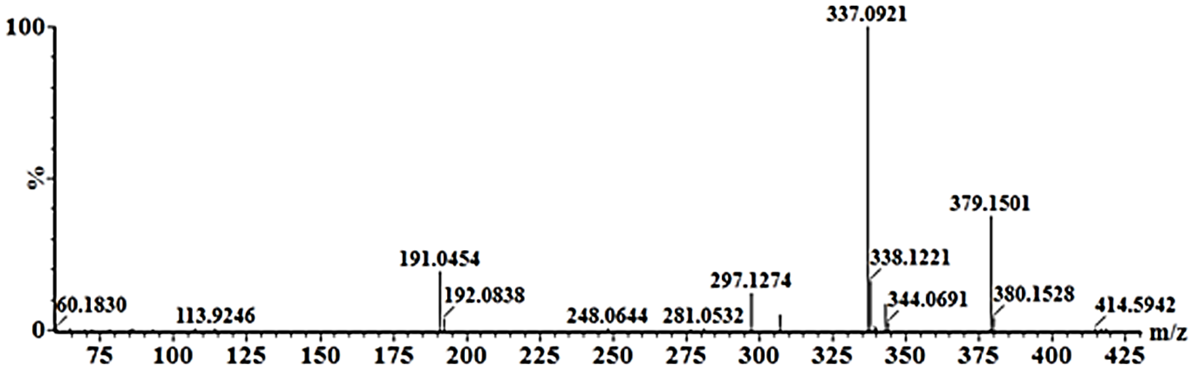

Figure S3: A typical mass spectrum of the fragmentation patterns of 5-coumaroylquinic acid.

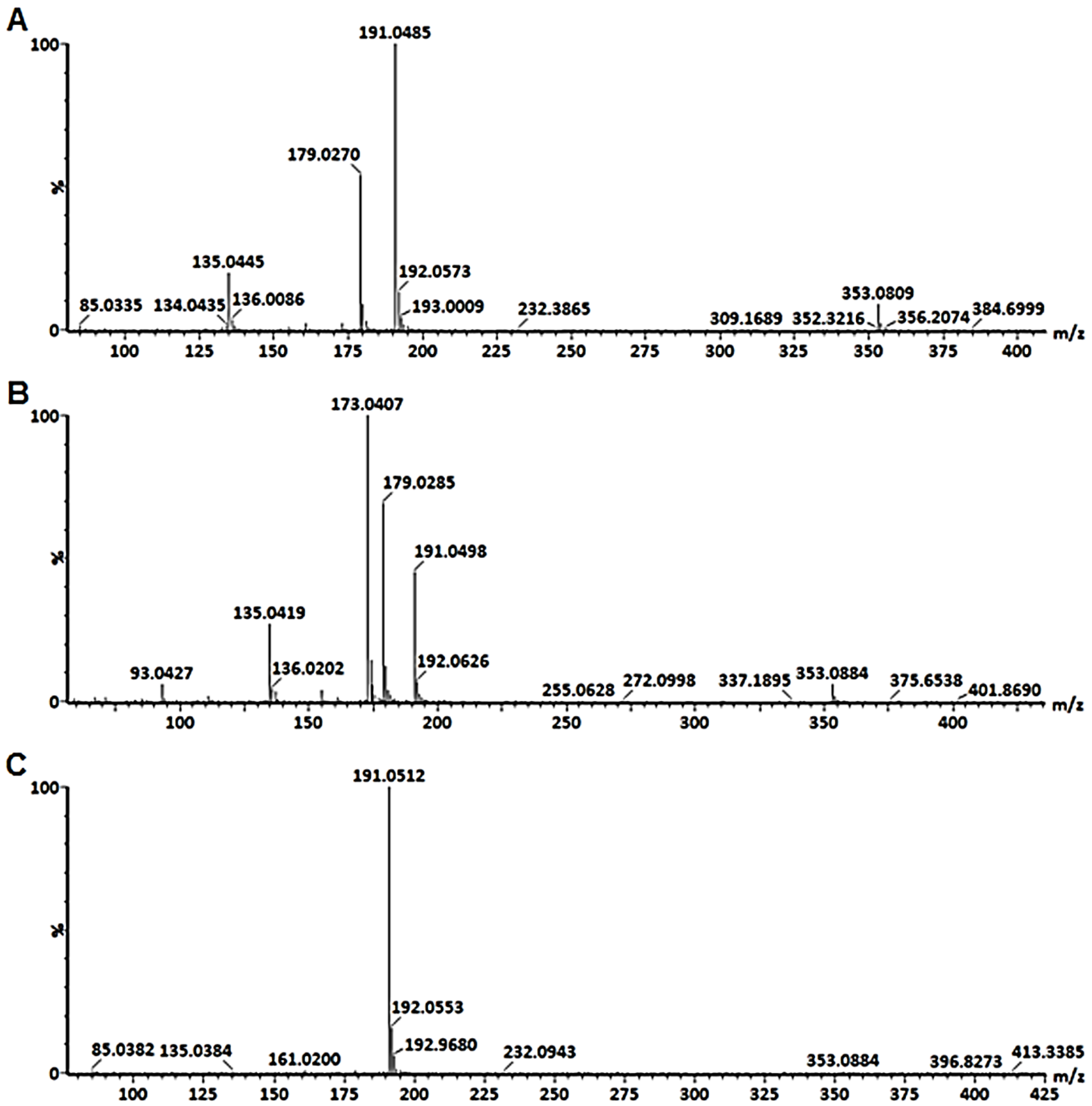

Figure S4: Typical mass spectra of the fragmentation patterns of 3-caffeoylquinic acid (A), 4-caffeoyquinic acid (B) and 5-caffeoylquinic acid (C).

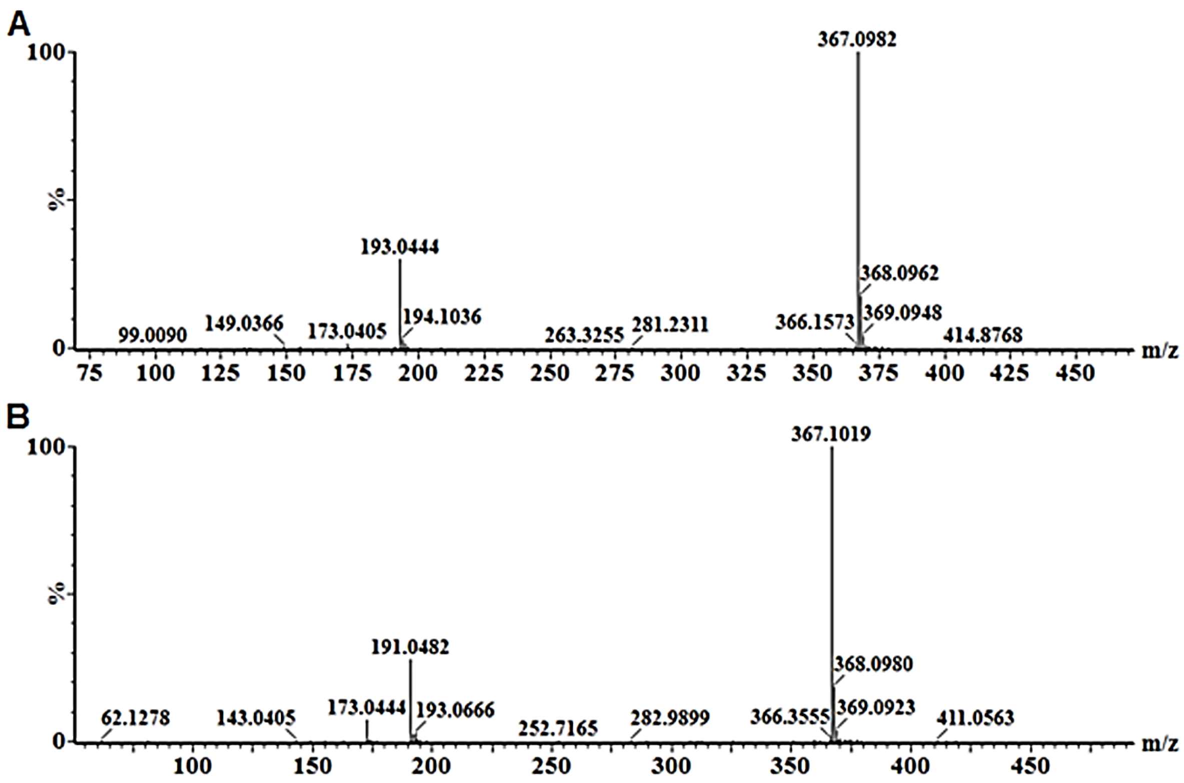

Figure S5: Typical mass spectra of the fragmentation patterns of 3-feruloylquinic acid (A) and 4-feruloylquinic acid (B).

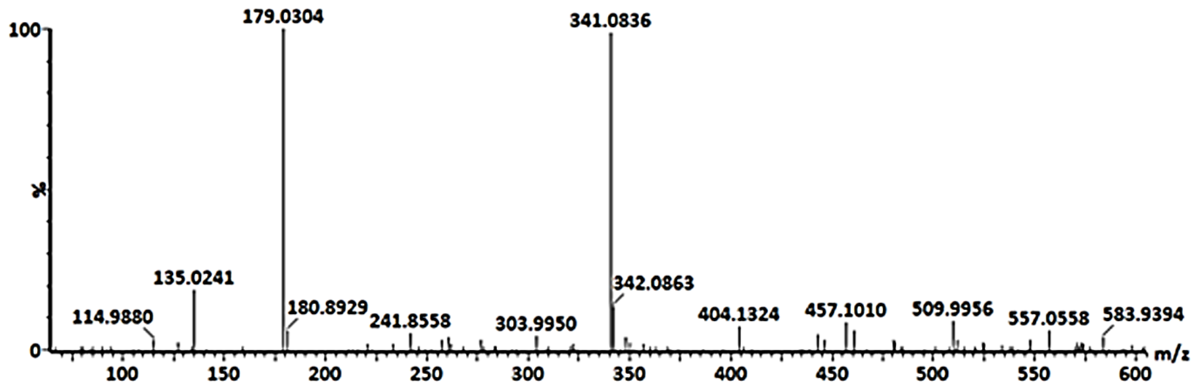

Figure S6: A typical mass spectrum of the fragmentation patterns of caffeoylglycoside.

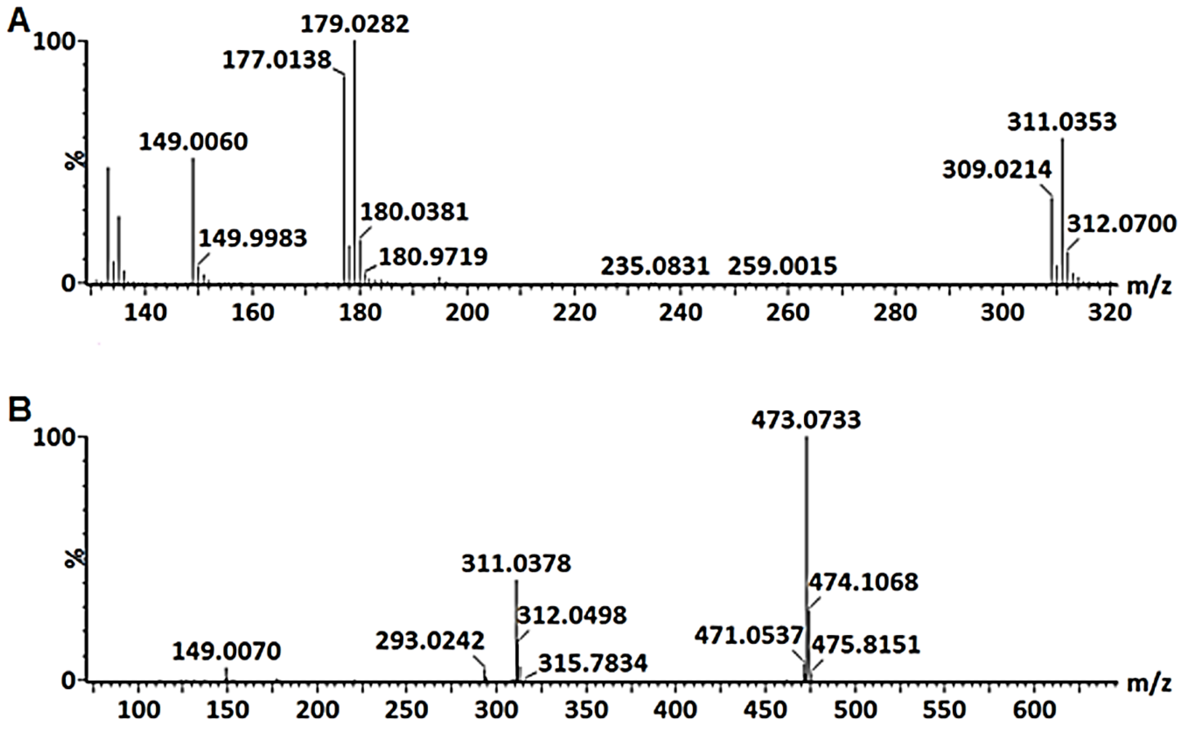

Figure S7: Typical mass spectra of the fragmentation patterns of caftaric acid (A) and chicoric acid (B).

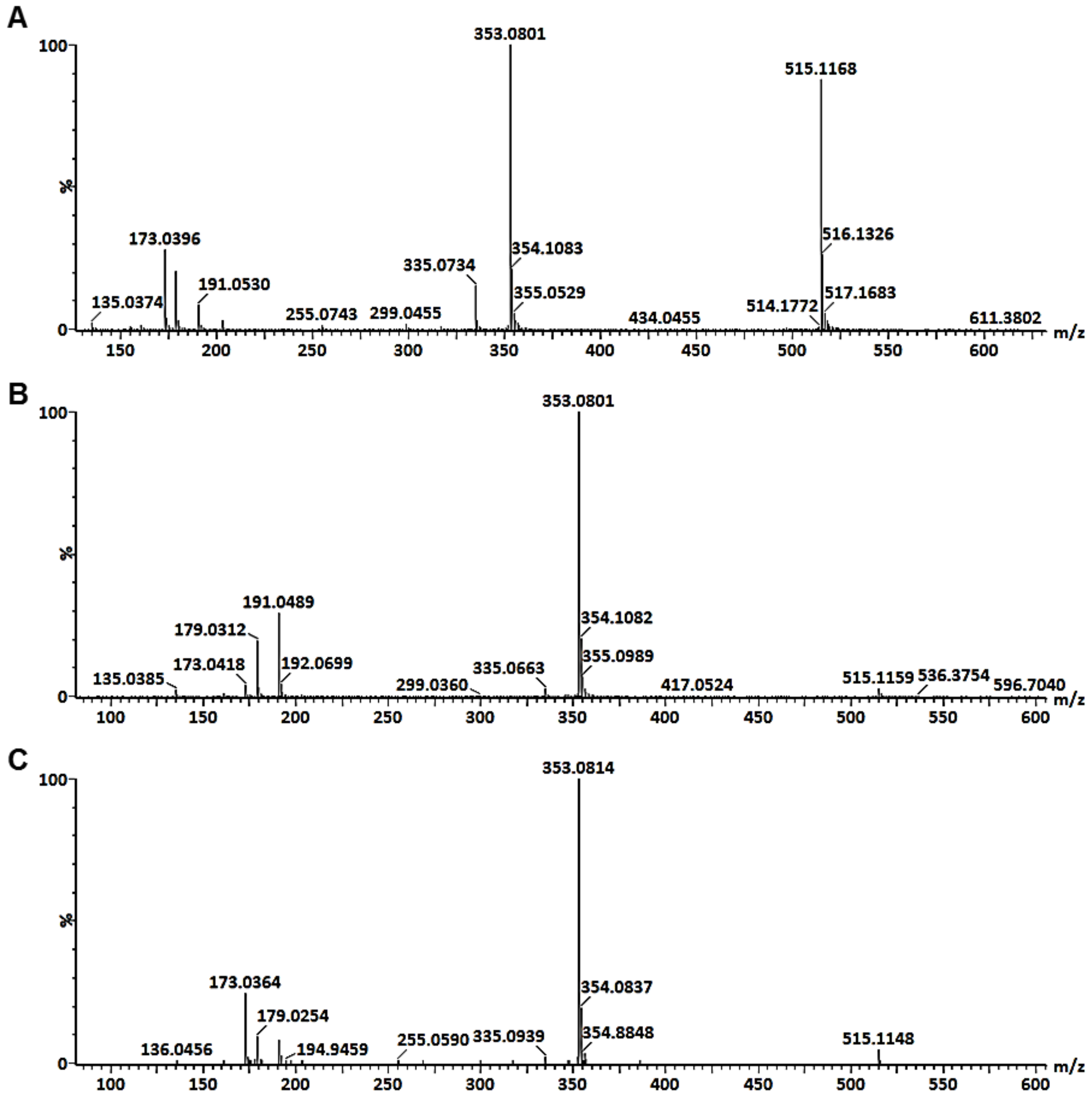

Figure S8: Typical mass spectra of the fragmentation patterns of 3,4-*di*-caffeoylquinic acid (A), 3,5-*di*-caffeoylquinic acid (B) and 4,5-*di*-caffeoylquinic acid (C).

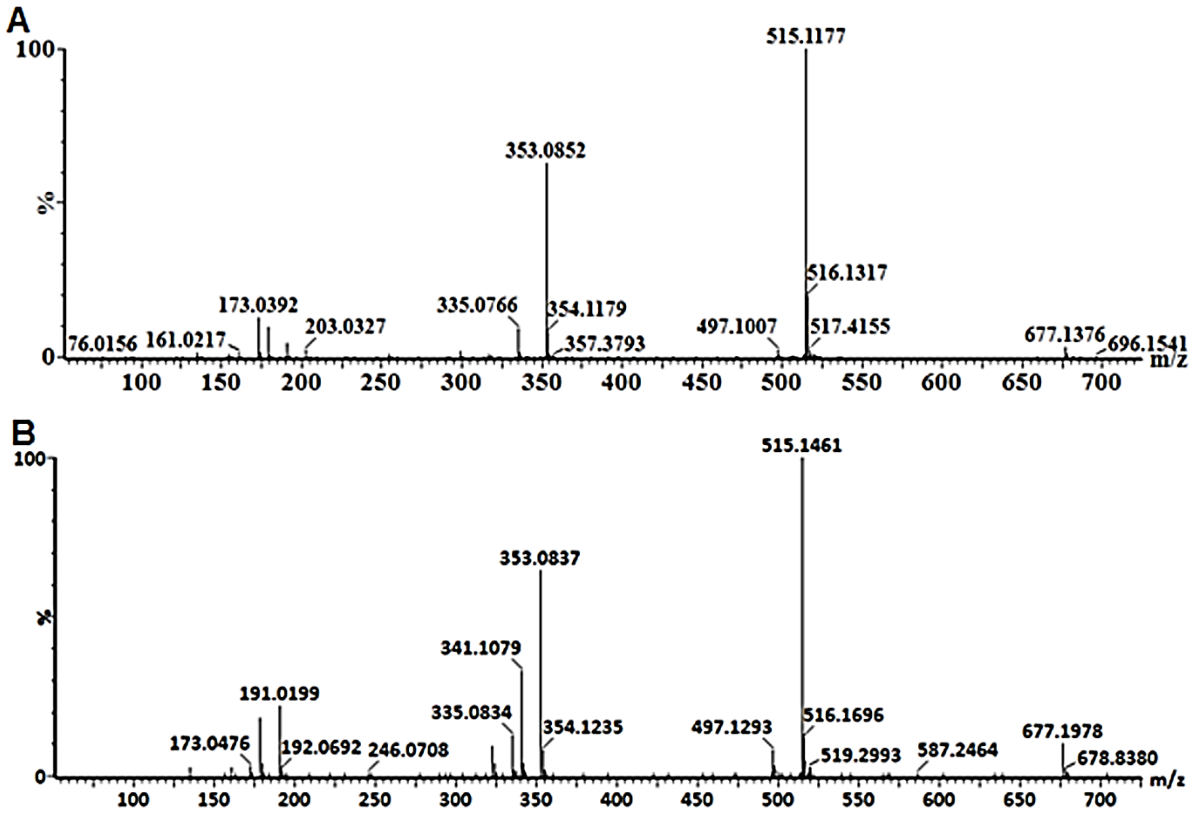

Figure S9: Typical mass spectra of the fragmentation patterns of *tri*-caffeoylquinic acid (A) and *di*-caffeoylquinic acid glycoside (B).
